# Feed‐Forward Deep Neural Networks Predict Substrate‐Specific Effects of Transporter Variants to Explain Drug Response Variability

**DOI:** 10.1111/cts.70592

**Published:** 2026-05-08

**Authors:** Yoomi Park, Yitian Zhou, Ming Xiao, Anne T. Nies, Volker M. Lauschke

**Affiliations:** ^1^ Department of Physiology and Pharmacology and Center for Molecular Medicine Karolinska Institutet and University Hospital Stockholm Sweden; ^2^ Medical Research Center Seoul National University College of Medicine Seoul South Korea; ^3^ Department of Information Science and Engineering Royal Institute of Technology, KTH Stockholm Sweden; ^4^ Dr. Margarete Fischer‐Bosch Institute of Clinical Pharmacology Stuttgart Germany; ^5^ University of Tübingen Tübingen Germany; ^6^ Cluster of Excellence iFIT (EXC 2180) “Image‐Guided and Functionally Instructed Tumor Therapies,” University of Tübingen Tübingen Germany; ^7^ Department of Pharmacy The Second Xiangya Hospital, Central South University Changsha China

**Keywords:** biobank, drug transporters, metformin, pharmacogenomics, precision medicine

## Abstract

Genetic variants in drug transporter genes shape the interindividual variability in drug response. However, their functional interpretation has remained limited due to the substrate dependence of variant effects. Existing predictors are substrate‐agnostic and cannot capture how a single amino acid change differentially affects transport across drugs. Here, we present the substrate‐specific effect predictor (SSEP), the first model to predict transporter variant effects in a substrate‐dependent manner. SSEP integrates curated in vitro uptake assays with deep mutational scanning data, leveraging multiscale features extracted from modeled variant–substrate complexes. Based on a feed‐forward deep neural network architecture, SSEP provides quantitative, substrate‐specific activity scores that correlate with experimental uptake data (Spearman's *ρ* = 0.64) across multiple transporter families (OCT1, MATE, CNT, and OATP) and substrate classes (biguanides, tetraethylammonium, selective serotonin agonists, sympathomimetics and opiates). In benchmarking analyses, SSEP showed higher concordance (Kruskal–Wallis *p* = 1.11 × 10^−41^) with experimental uptake than commonly used substrate‐agnostic predictors. Application of SSEP on UK Biobank data revealed that OCT1 (*SLC22A1*) variant burden weighted by metformin‐specific SSEP scores was significantly associated with maintenance dose (*β* = 30 mg/day per unit increase in predicted functional burden; *p* = 0.033), whereas substrate‐agnostic weights showed no association (*p* = 0.78). Together, these results show that SSEP enables quantitative, drug‐specific prediction of transporter variant effects, thereby improving the identification of clinically relevant transporter variants in population‐scale data.

## Study Highlights

- What is the current knowledge on the topic?
  ○ Genetic variants in drug transporters contribute substantially to interindividual variability in drug response but can exert strongly substrate‐dependent effects.
  ○ Existing computational variant effect predictors are substrate‐agnostic and cannot capture this heterogeneity, complicating clinical translation.
- What question did this study address?
  ○ Can transporter variant effects be predicted in a substrate‐specific manner, and does incorporating substrate specificity improve functional interpretation and clinical relevance compared with conventional substrate‐agnostic approaches?
- What does this study add to our knowledge?
  ○ This study introduces the substrate‐specific effect predictor (SSEP), a machine‐learning framework for predicting transporter variant effects in a substrate‐specific manner.
  ○ By integrating experimental uptake data and multiscalar biochemical and sequence features, SSEP enables quantitative, drug‐specific prediction of variant function, including for variants lacking direct experimental characterization.
  ○ This approach reveals clinically relevant associations between OCT1 variants and metformin maintenance dose that were not detected using nonspecific scores.
- How might this change clinical pharmacology or translational science?
  ○ By enabling drug‐specific interpretation of transporter variants, SSEP enables incorporation of substrate‐specificity into pharmacogenomic analyses.
  ○ Substrate‐specific predictions can improve genotype‐guided dosing strategies and better support the stratification of patients according to drug response.
  ○ In early drug development, SSEP may assist in assessing transporter liability and variant sensitivity for novel substrates.

## 1 Introduction

Advances in population pharmacogenomics have revealed extensive genetic variation in drug transporters, alongside rapidly growing datasets linking genetic diversity to clinical outcomes [1, 2]. Population‐scale genomic resources have catalogued millions of variants across diverse ancestries, while biobank initiatives provide linked prescription and genetic data for hundreds of thousands of participants. In parallel, rapid progress in structural biology and computational modeling has yielded high‐resolution transporter structures [3]. Together, these developments provide unprecedented opportunities to systematically dissect the functional consequences of transporter variants at scale.

Despite this progress, the functional interpretation of transporter variants has remained fragmentary. A few transporters, such as OATP1B1 (encoded by *SLCO1B1*) and BCRP (*ABCG2*), are included in current pharmacogenetic guidelines and supported by well‐established functional and clinical evidence [4]. However, for most others, including the clinically relevant solute carriers OCT1 (*SLC22A1*), MATE1/2 (*SLC47A1*/*2*), and CNT3 (*SLC28A3*), reported variant effects remain scattered across studies and often yield conflicting results [5]. A major reason for this uncertainty is the pronounced substrate specificity of transporter variant effects [6]. For example, the common OCT1 variant p.M420del reduces uptake of metformin, morphine, tropisetron, and *O*‐desmethyltramadol by more than 75%, yet shows near‐normal activity for other drugs such as sumatriptan or debrisoquine [7, 8]. Similarly, OCT1 p.F244A reduced uptake of some substrates, such as deoxyepinephrine, by up to 10‐fold, while increasing uptake for pirenzepine by 8‐fold [9]. Such highly divergent, substrate‐dependent effects cannot be adequately captured by conventional algorithms that produce only binary variant effect predictions, complicating translation of genomic findings into clinical interpretation.

Numerous computational tools have been developed to predict variant effects, including SIFT, PolyPhen‐2, CADD, and more recent deep learning models such as AlphaMissense [10, 11, 12, 13]. While effective for identifying globally disruptive mutations, these tools are inherently substrate‐agnostic, relying primarily on sequence conservation and structural stability rather than ligand‐dependent interactions. As a result, they cannot capture the heterogeneous, substrate‐dependent functional effects that underpin transporter pharmacogenetics. Although curated uptake datasets exist, they cover only a small subset of naturally occurring variants, and emerging deep mutational scanning (DMS) resources, while broader in coverage, typically use only one or a few reporter substrates and therefore lack substrate‐specific resolution.

To address these challenges, we developed the Substrate‐Specific Effect Predictor (SSEP), designed to predict the functional impact of transporter variants in a substrate‐specific manner. The current study focuses on solute carrier (SLC) transporters, primarily *SLC22* (OCT) and the related families *SLC6*, *SLC47* (MATE), *SLC28* (CNT), and *SLCO* (OATP). Our approach integrates curated experimental uptake assays with currently available transporter DMS datasets for OCT1 (*SLC22A1*) and SERT (*SLC6A4*) to learn both general and substrate‐dependent patterns of variant activity. Structural modeling and ligand docking were used to construct variant–substrate complexes, from which multiscale features were extracted. This design enables prediction of quantitative, substrate‐specific functional effects for both single‐nucleotide and indel variants across transporters with available structural models. The resulting SSEP showed strong concordance with experimental uptake data across multiple transporter families and identified clinically relevant substrate‐specific associations between OCT1 variants and metformin maintenance dose in the UK Biobank, which were not identified when using substrate‐agnostic scores. Together, SSEP provides a transferable framework for predicting substrate‐specific functional effects across diverse drug transporters.

## 2 Materials and Methods

### 2.1 Data Collection

We compiled relative uptake activity data for transporter variants from published studies [7, 8, 14, 15, 16, 17, 18, 19, 20, 21, 22, 23, 24, 25, 26, 27, 28, 29, 30, 31]. When multiple measurements were available for the same variant–drug pair, the mean uptake ratio relative to wildtype was calculated. The dataset does not include kinetic parameters (e.g., *V*_max_ or *K*_m_), and no averaging across concentrations or experimental conditions was performed. For model development, the well‐characterized OCT1 p.F244A variant, known for its substrate‐specific effects (136 p.F244A‐substrate pairs), together with in vitro functionality data for 36 transporter variant–drug pairs spanning eight transporter genes used in our earlier work [32], were included as the pretraining set (Table S1). The remaining 635 variant–drug pairs were reserved for independent evaluation to ensure unbiased model assessment (Table S2). All publicly available human transporter DMS datasets (i.e., OCT1 [33] and SERT [34]) were obtained from ProteinGym [35] and were used exclusively for model fine‐tuning.

### 2.2 Mutant Structure Modeling and Ligand Docking

Primary ligand structures were retrieved from PubChem [36] or curated manually when automatic resolution was not possible. Experimentally determined transporter structures were obtained from PDB (Table S3). When multiple structures were available, nonredundant high‐quality chains were retained. When a cocrystal ligand was unavailable, wild‐type complexes were generated using AutoDock Vina (v1.1.2). Missense and indel variants were built on the wild‐type template using homology‐based approaches. When experimental PDB coverage was missing at the variant site or a gene had no suitable PDB entry, AlphaFold2‐derived [37] templates were used. Ligands were docked into the predicted binding pockets, and resulting complexes used for downstream feature extraction.

### 2.3 Feature Extraction

All extracted features are shown in Table S4. Detailed steps and full feature definitions are described in the Supporting Information. In short, sequence‐level features were derived from mutant sequences, for which embeddings were generated using the pretrained ESM‐2 language model [38]. Variants were annotated with established in silico prediction scores. Residue‐level physicochemical properties and predicted stability changes were computed for each mutation. Ligand and protein‐ligand interaction features were calculated from docked complexes, including distance‐based interaction metrics capturing the local binding environment. Binding‐site residues were defined based on proximity to the ligand.

### 2.4 Hyperparameter Optimization and Machine‐Learning Model Construction

We built two substrate‐specific effect prediction models: the substrate‐specific effect predictor for single nucleotide variants (SSEP‐SNV) with complete annotations and the substrate‐specific effect predictor for universal variants including indels (SSEP‐UV) for variants lacking full feature coverage, including indels. Both models were implemented as multi‐input feed‐forward deep neural networks (DNNs) integrating sequence embeddings, evolutionary scores, mutation biophysical descriptors, ligand physicochemical descriptors, and local structural environment features. Models were trained using the Adam optimizer and mean squared error (MSE) loss. Hyperparameter optimization was performed on the pretraining dataset using grid search. Because rank‐preserving predictions are most relevant for pharmacogenomic applications, model performance was assessed using Spearman correlation between predicted and observed uptake values. The optimal hyperparameter set was selected based on average ρ across repeated runs (Figure S1). The final model was retrained on the entire pretraining dataset. When multiple structural models were available for the same variant–substrate pair, predictions were averaged to obtain a single SSEP score.

### 2.5 Model Application Using UK Biobank

Primary care prescription records from the UK Biobank were used to derive metformin‐specific maintenance doses [39]. Prescriptions were harmonized, filtered for valid dosing information, and converted to average daily dose. Associations between OCT1 variant burden and metformin maintenance dose were evaluated using linear regression. Analyses were restricted to variants with a minor allele frequency (MAF) < 0.05 in the non‐Finnish European (NFE) cohort of gnomAD. For each individual, a variant burden score was calculated as the sum of allele dosages weighted by either metformin‐specific scores or the mean in vitro activity across all OCT1 substrates. Association with maintenance dose was assessed using linear regression adjusted for age at prescription, sex, ethnic group, and body mass index (BMI). For rare‐variant aggregation tests, damaging variants were defined according to SSEP and the damaging thresholds of a panel of 19 established in silico variant effect predictors. Two complementary gene‐level association tests, SKAT‐O and burden testing, were implemented.

### 2.6 Feature Importance Analysis

To identify the most informative predictors in the pretraining dataset, we first reduced redundancy by removing collinear variables. Because the most influential predictive features are high‐dimensional embeddings that are not directly interpretable, this analysis was designed as a descriptive exploration of the labeled training data rather than a model‐based feature attribution. Pairwise Spearman's correlations were calculated, and for feature pairs with |*ρ*| > 0.9, only one feature was retained. The remaining features were then tested for association with substrate‐specific activity using non‐parametric Kruskal–Wallis tests, grouping variant–substrate pairs into decreased (< 50% relative uptake), neutral, or increased (> 200%) categories. Significance values were adjusted using the Benjamini–Hochberg false discovery rate, and features were ranked accordingly. The four most discriminative features were selected for downstream analysis.

## 3 Results

### 3.1 Transporter Variants Exhibit Substrate‐Specific Functional Diversity

**FIGURE 1:**
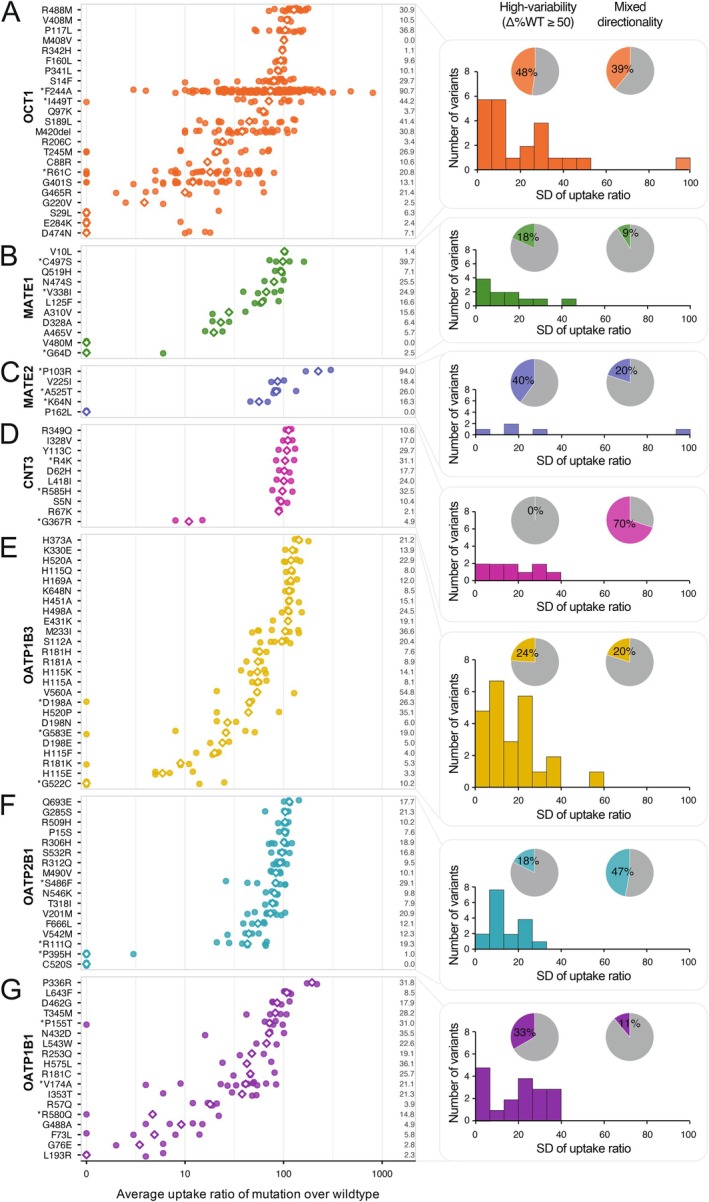
Substrate‐specific uptake ratios of transporter mutations. The *x*‐axis shows the average mutant‐to‐wildtype uptake ratio, plotted on a log‐scale (log10[1 + *x*]) for variants in OCT1 (A), MATE1 (B), MATE2‐K (C), CNT3 (D), OATP1B3 (E), OATP2B1 (F), and OATP1B1 (G). Points represent variant‐substrate pairs. Only variants are shown for which data for at least two substrates was available. Within each panel, mutations are ordered by their median uptake ratio (white diamonds). Numbers to the right of each row show the standard deviation of uptake ratios across substrates. Variants marked with an asterisk denote the three mutations with the highest substrate‐dependent span (maximum/minimum uptake ratio) within each transporter. To the right of each dot plot, pie charts summarize, for each transporter, (i) the percentage of variants showing high substrate‐dependent variability, defined as a within‐variant variability span of ≥ 50 (Δ%WT = max[%WT] − min[%WT]; middle‐left panels) and (ii) the percentage of variants exhibiting mixed directionality (red), defined as variants that show both increased (uptake ratio > 100) and decreased (< 100) activity across substrates (middle‐right panels, red). Adjacent histograms display the distribution of standard deviations for all variants within each gene (right panels). Each summary panel corresponds to the respective transporter (A–G).

To develop a model for the prediction of substrate‐specific transporter pharmacogenomics, we first parsed the substrate variability of transporter variants reported in the literature. Functional profiling across seven clinically important transporters revealed substantial diversity in variant effects on substrate uptake (Figure 1). OCT1 variants exhibited the largest substrate variability in their activity profiles with 48% of variants (11/23) exhibiting high variability defined as within‐variant activity span across substrates ≥ 50% (Δ%WT ≥ 50). The aromatic substitution p.F244A showed some of the highest substrate‐specific effects, with uptake ranging from 10% of wildtype activity to an 8‐fold increase (SD = 90.7; Figure 1A). The common indel p.M420del also demonstrated divergent effects, showing reduced activity for metformin and morphine but retaining or even increasing uptake for sumatriptan. The variant p.R61C showed substrate‐dependent transport activity, with comparatively higher relative uptake of MPP+ and cycloguanil than of metformin and thiamine. In contrast, p.R488M exhibited increased uptake of metformin and thiamine but showed reduced transport of debrisoquine. These examples illustrate that the functional consequences of OCT1 variants can vary substantially across substrates.

For MATE1, MATE2‐K and CNT3, the substrate specificity of variants was generally lower (Figure 1B–D). Only 18% of MATE1 variants exhibited Δ%WT ≥ 50 and 9% showed mixed directionality, reflecting largely uniform effects. In contrast, a higher proportion of high‐variability variants were identified for MATE2‐K (40%), though these estimates should be interpreted cautiously given the relatively smaller number of variants analyzed. Some variants, such as MATE2‐K p.P103R, showed very high albeit unidirectional substrate variability (SD = 94), indicating large quantitative but not qualitative differences among substrates. CNT3 displayed the highest proportion of mixed‐direction variants (70%), despite none exceeding the three‐fold variability threshold, suggesting that most uptake values fluctuated only modestly around the wild‐type level. In comparison, the OATP family exhibited consistently broad functional heterogeneity, with 18%–33% of variants displaying high variability across substrates and 11%–47% showing mixed directionality (Figure 1E–G). For example, OATP1B1 p.V174A, a well‐established pharmacogenetic variant [40], showed reduced activity for rifampin and rosuvastatin, but preserved near‐normal transport for estrone‐3‐sulfate. Similar substrate‐specific patterns where certain variants caused pronounced functional impairment for one compound yet had little or no effect on others were observed for OATP1B3 and OATP2B1. Together, these findings show that the functional effects of transporter variants cannot be adequately captured by a single, substrate‐agnostic value.

### 3.2 Development of a Framework for the Substrate‐Specific Effect Prediction of Transporter Variants

**FIGURE 2:**
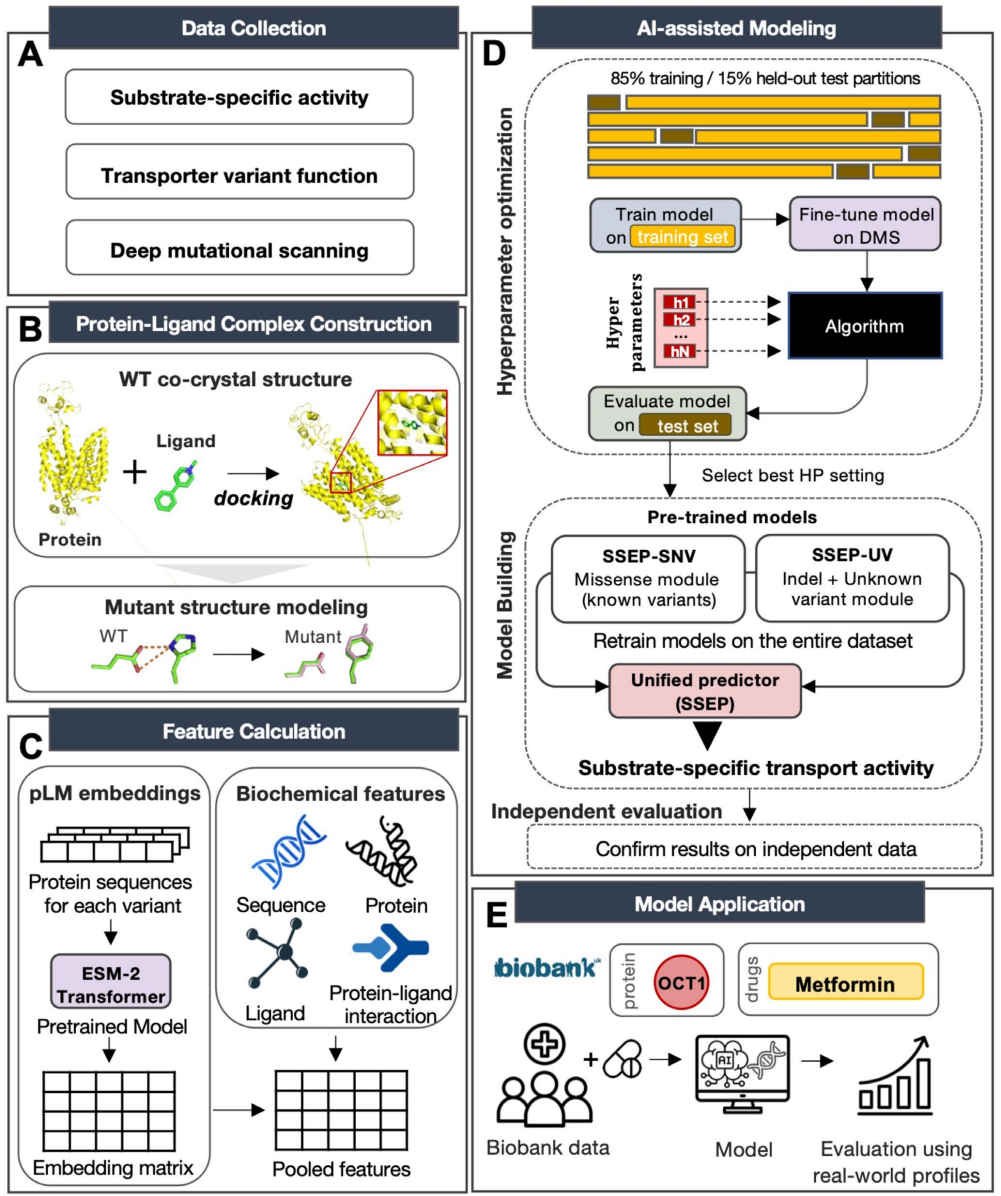
SSEP development workflow. (A) Curated experimental activity ratios (mutant/wildtype) were compiled for 172 variant–drug pairs, incorporating both substrate‐specific activity and transporter function data for pretraining, with 635 independent sets reserved for evaluation. Deep mutational scanning (DMS) data for human transporters from ProteinGym were used for model fine‐tuning. (B) Native cocrystal structures were obtained from the Protein Data Bank, and mutant structures were generated from these wildtype templates. Target ligands were docked into binding sites using Autodock Vina. (C) Protein language model (pLM) embeddings were derived using the pretrained ESM‐2 transformer and complemented with biochemical features from protein, ligand, and environmental descriptors. (D) Models were trained using an 85%–to‐15% train‐test split, fine‐tuned on DMS data, and optimized through hyperparameter (HP) selection. Two modules were trained separately: SSEP‐SNV for missense with complete annotations and SSEP‐UV for indels and novel/unknown variants. After HP selection, each module was retrained on the entire training set. For each variant–drug pair, the module outputs were integrated to yield a single SSEP activity score. Performance was evaluated on an independent dataset (176 variants from 8 transporters). (E) The final model was applied to Biobank data, where predictions were evaluated against drug response profiles.

To address this gap, we established a computational framework to predict the functional effects of transporter variants in a substrate‐specific manner. Specifically, we used a machine learning approach in which the model was trained on experimentally measured uptake activity ratios (mutant/wildtype) from transporter variant–drug pairs. For learning, we used a total of 172 variant–substrate pairs from nine SLC transporter genes (Figure 2A). An additional independent set of 635 variant–drug pairs was reserved exclusively for model evaluation to prevent information leakage. To increase variant coverage and improve generalizability, we further integrated DMS datasets of OCT1 (*SLC22A1*) and SERT (*SLC6A4*) for model fine‐tuning. Although DMS assays were performed with a single reporter substrate, incorporating these scores during fine‐tuning enabled the model to learn general transporter activity while maintaining sensitivity to substrate‐specific effects observed in multi‐substrate uptake assays. Consequently, DMS fine‐tuning substantially increased model performance (Figure S2). Together, these complementary activity profiles aimed to enable the model to capture both general variant effects and substrate‐specific dimensions of transporter variant function.

To allow for quantitative modeling of transporter variant effects, we constructed a unified structural framework comprising wildtype and mutant protein–ligand complexes (Figure 2B). Experimentally determined substrate‐bound cocrystal structures were used whenever available. Otherwise, molecular docking was performed to predict the most energetically favorable substrate poses. Mutant complexes were subsequently generated from wildtype templates, allowing for the systematic comparison of residue‐specific perturbations on the binding environment. From these complexes, we extracted comprehensive multiscalar feature sets capturing both sequence‐ and structure‐level information (Figure 2C). This included biochemical and structural mutation descriptors, ligand physicochemical properties, local protein–ligand interaction features, and in silico prediction scores from general variant effect predictors. In parallel, embeddings from the pretrained ESM‐2 protein language model were incorporated to represent nonlocal relationships between amino acids and evolutionary constraints.

This integrated dataset provided the basis for AI‐assisted modeling (Figure 2D). When the complete feature set was available for a variant, the SSEP‐SNV module was used, whereas SSEP‐UV extended predictions to variants lacking full annotations. These efforts resulted in a single SSEP score for each variant‐substrate pair. In total, 1478 missense variants from gnomAD were mapped onto OCT1 and SERT, of which 1387 (93.8%) had complete annotations and were retained for modeling, demonstrating that SSEP‐SNV captures the majority of naturally occurring variants in these transporters. Finally, we evaluated the real‐world applicability of SSEP by applying the model to human clinicogenetic data from the UK Biobank (Figure 2E). Together, integration of these orthogonal datasets by the SSEP framework enabled quantitative, substrate‐specific prediction of transporter variant effects across structurally resolved transporters.

### 3.3 Evaluation of SSEP Predictive Accuracy

**FIGURE 3:**
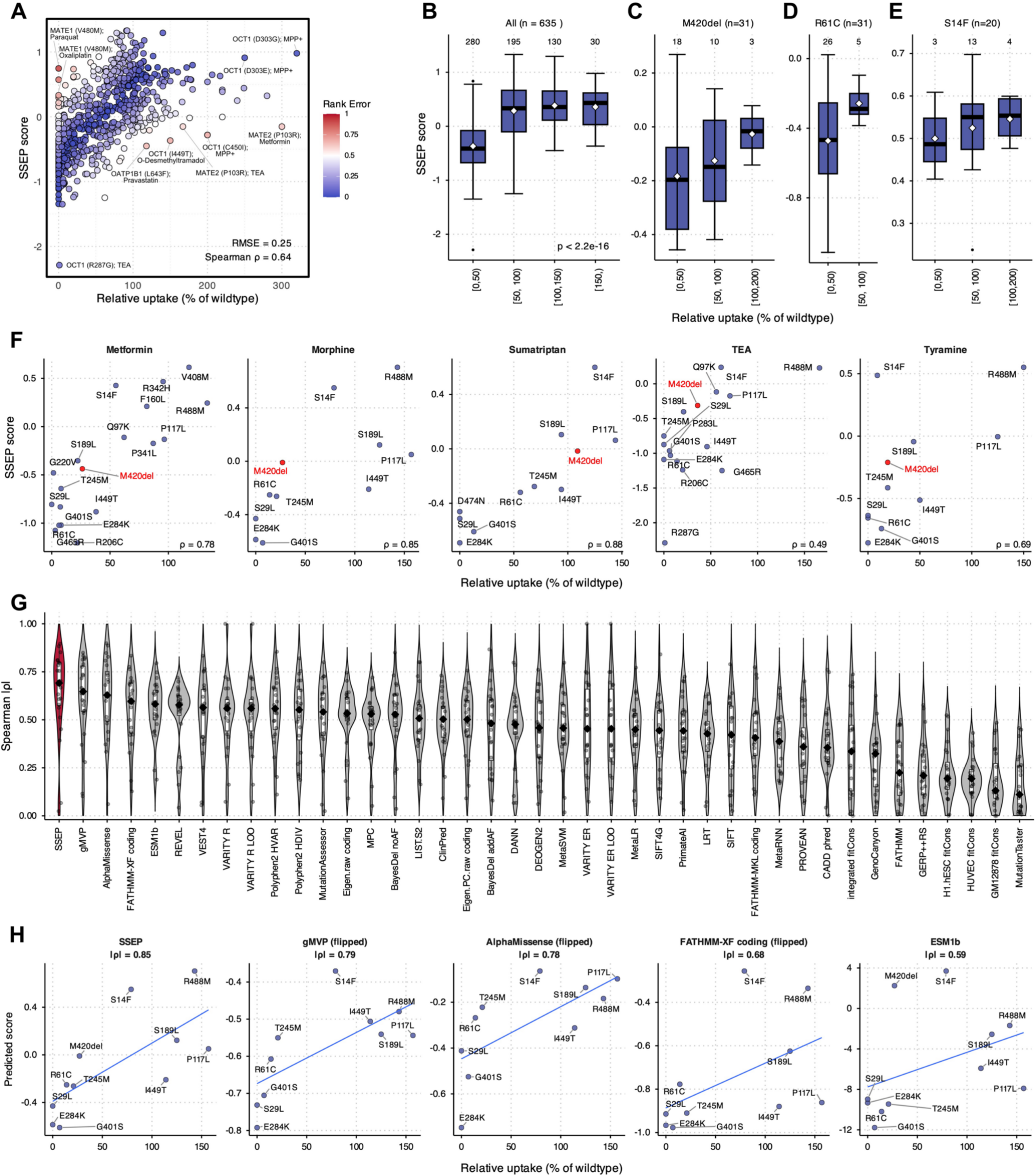
Performance and score distribution of SSEP on independent in vitro data. (A) Observed vs. predicted for individual variant‐substrate pairs. The *x*‐axis shows observed relative uptake of the mutant compared to wildtype and the *y*‐axis shows the average SSEP score (mean across all available PDB‐derived complexes for that pair). Points are colored by rank error, with blue indicating lower error and red indicating higher error. Model performance is summarized by the root mean square error (RMSE) and Spearman's correlation coefficients (*ρ*). The distribution of SSEP scores is shown across binned transporter functionality for all variant–substrate pairs (B), as well as for OCT1 p.M420del (C), p.R61C (D), and p.S14F (E), as variants with ≥ 20 distinct drug pairs. Numbers above each box indicate the count of unique variant–drug pairs in that bin. White diamonds mark the mean SSEP score. Jonckheere's trend test *p*‐values are shown. (F) Correlations between SSEP scores and experimental uptake are shown for five drugs with each point representing a variant–drug pair. Spearman's correlation coefficients (*ρ*) between predicted and measured activity are shown for each drug. (G) Violin plots of per‐substrate absolute Spearman's correlations (|*ρ*|) between experimental uptake and 40 substrate‐agnostic predictors across substrates with ≥ 10 variants. (H) Scatterplots for morphine as an example comparing SSEP with the top four other algorithms (gMVP, AlphaMissense, FATHMM‐XF coding, ESM1b). Regression lines are shown in blue.

We first evaluated the prediction performance of SSEP using an independent set of in vitro uptake measurements (176 variants from 8 transporters; Figure 3A). SSEP generates continuous prediction scores in the range from −2.29 to +1.33, reflecting the relative functional impact of variants on substrate transport. Lower values indicate reduced transport activity while higher values signal preserved or increased activity relative to the reference allele. Predicted activities showed strong correlation with experimentally measured uptake values (Spearman's ρ = 0.64, *p* = 4.62 × 10^−62^; root mean square error [RMSE] = 0.25). Visualization of variant‐substrate pairs by rank error showed that, while non‐OCT1 variants exhibited greater dispersion (RMSE = 0.26–0.41 vs. 0.22 for OCT1), the majority of predictions closely followed the experimental trend, indicating that the model accurately captured the quantitative magnitude of substrate‐specific functional effects. To further evaluate substrate specificity, we compared SSEP scores across activity bins defined by relative uptake. Across all 635 variant‐substrate pairs, predicted scores significantly correlated with experimental activity (*p* < 2.2 × 10^−16^; Figure 3B). Similar trends were observed at the individual variant level. For example, the common deletion variant OCT1 p.M420del showed SSEP scores that closely mirrored its experimentally measured uptake across substrates (Figure 3C). The disruptive variant p.R61C consistently exhibited lower predicted scores (Figure 3D), while p.S14F retained higher predicted values overall, consistent with its preserved global activity but also displaying substrate‐dependent variation (Figure 3E).

To evaluate whether SSEP captures both variant functionality and substrate specificity, we compared model predictions with in vitro uptake measurements. For this analysis, we focused on five substrates of OCT1 due to the broad availability of variant‐substrate transport activity data. Importantly, predicted SSEP scores showed strong positive correlations with experimentally measured uptake (Figure 3F, *ρ* = 0.78 for metformin, *ρ* = 0.85 for morphine, *ρ* = 0.88 for sumatriptan, *ρ* = 0.49 for tetraethylammonium [TEA], and *ρ* = 0.69 for tyramine), indicating that the model accurately captured quantitative variant activities for each substrate. SSEP also reflected the substrate‐specific impacts of variants on transport activity. For instance, the common indel variant p.M420del showed markedly reduced uptake of metformin but retained or exceeded wildtype activity for sumatriptan, and SSEP correctly reproduced this directional shift.

Finally, we benchmarked SSEP against commonly used substrate‐agnostic variant effect predictors, by comparing the correlation between experimentally measured uptake and prediction scores across all substrates that contained at least 10 variants. The median absolute Spearman's correlation (|*ρ*|) for SSEP was the highest among the tested methods (Kruskal–Wallis *p* = 1.11 × 10^−41^), highlighting the importance of substrate‐specific modeling to reproduce quantitative variant activities (Figure 3G). Using morphine as a representative substrate with well‐established OCT1 transport, we found that SSEP scores closely tracked experimental uptake values (|*ρ*| = 0.85), whereas the correlations for generic predictors were weaker (gMVP |*ρ*| = 0.79, AlphaMissense |*ρ*| = 0.78, FATHMM‐XF |*ρ*| = 0.68, and ESM1b |*ρ*| = 0.59; Figure 3H). Together, these findings demonstrate that SSEP captures both the quantitative functionality of variants within individual substrates and their substrate‐specific variability across different drugs.

**FIGURE 4:**
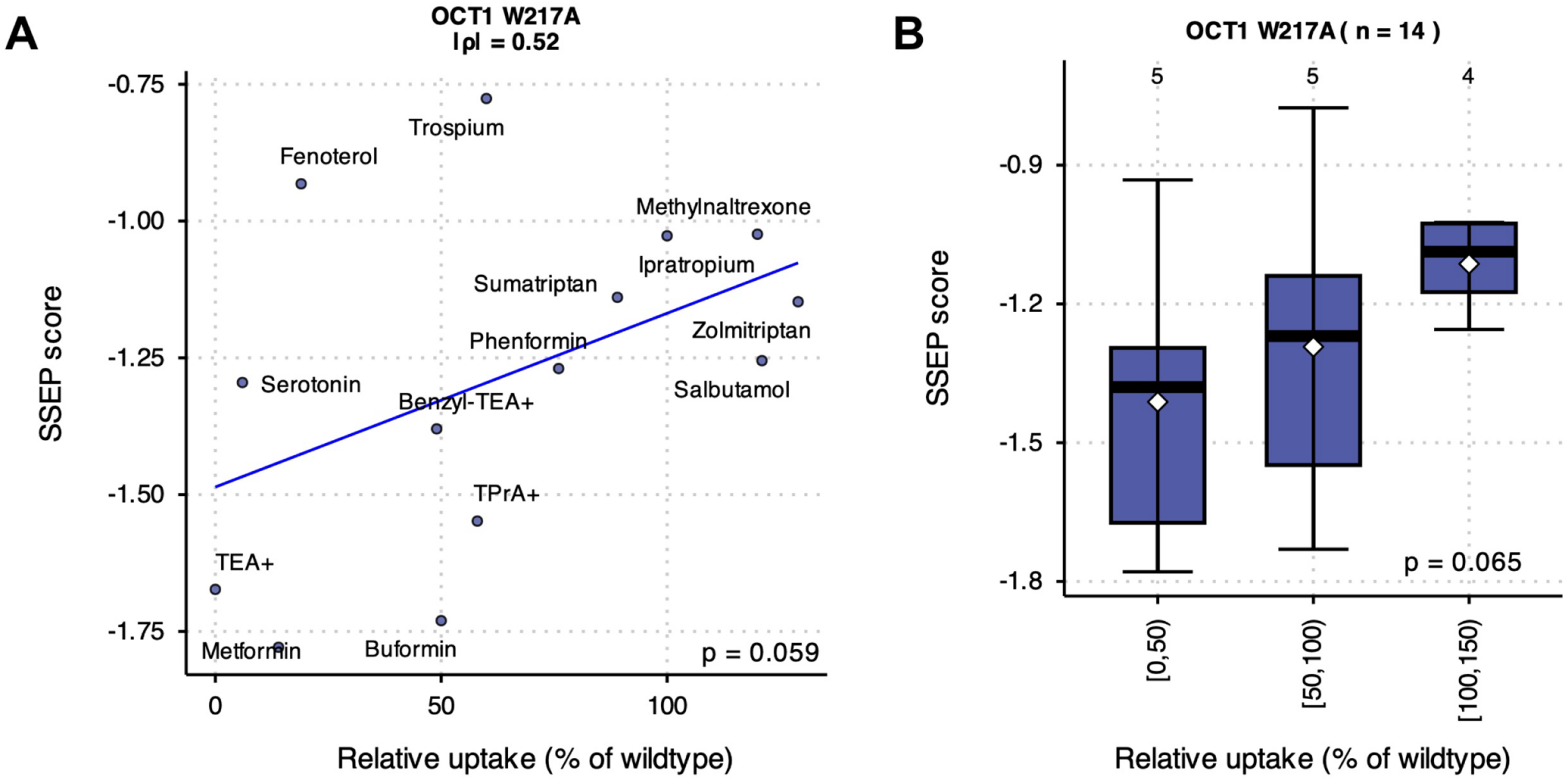
External in vitro evaluation of SSEP using OCT1 p.W217A. (A) Correlation between experimentally measured relative uptake (% of wildtype) and SSEP prediction scores for the OCT1 p.W217A variant across 14 substrates. Association was assessed using Spearman's rank correlation. (B) Distribution of SSEP scores stratified by experimentally measured uptake bins. Boxes show median and interquartile range; whiskers indicate range; white diamonds denote means. Numbers above boxes indicate the number of substrates per bin. Monotonic trends across uptake bins were evaluated using the Jonckheere–Terpstra trend test.

While the above analyses establish the global predictive performance of SSEP, we next evaluated model transferability. To this end, we leveraged a recent dataset describing substrate‐specific effects of OCT1 p.W217A [41]. Using this independent validation set, we found that SSEP predictions were positively correlated with experimentally measured uptake values (Spearman's ρ = 0.52, *p* = 0.059) across 14 OCT1 substrates (Figure 4A). When stratified by uptake bins, predicted scores showed a consistent trend across activity categories (Figure 4B). These findings demonstrate that SSEP reproduces both overall variant‐level activity trends and within‐variant heterogeneity across substrates, supporting more general applicability for modeling substrate‐specific transporter variant effects.

### 3.4 Classification Performance of Substrate‐Specific Variant Effect Predictions

**FIGURE 5:**
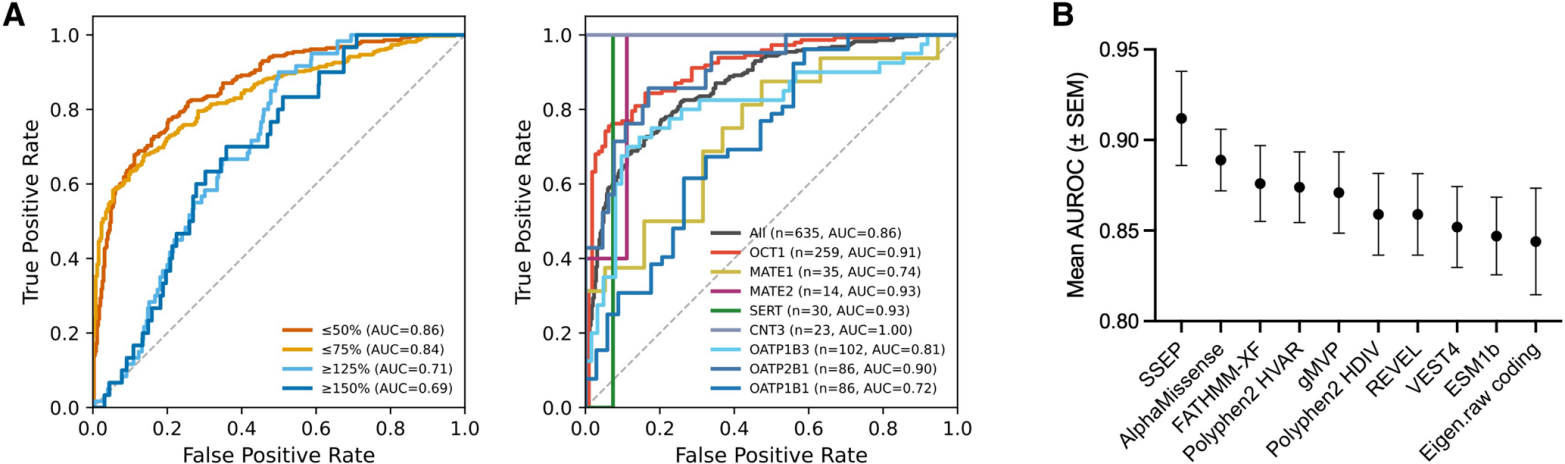
SSEP performance on the independent evaluation set. (A) Receiver operating characteristic (ROC) curves for SSEP. The left panel shows the area under the ROC curve (AUC_ROC_) at threshold levels of 50%, 75%, 125%, and 150% of uptake ratios. The right panel shows AUC_ROC_ by protein, with uptake ratios < 50% of wildtype considered indicative of reduced function. (B) Mean AUC_ROC_ (± SEM) across substrates for the SSEP and the nine in silico prediction scores with the highest average AUC_ROC_. For each substrate, AUC_ROC_ was computed on the corresponding variant–substrate pairs. Per‐substrate AUC_ROC_ was then averaged across substrates with ≥ 10 variants to summarize mean performance.

We next evaluated the predictive performance of SSEP in a classification framework. Receiver operating characteristic (ROC) analyses showed high overall accuracy across a range of activity thresholds. SSEP showed the highest discriminative power for reduced function variants with areas under the ROC curve (AUC_ROC_) of 0.86 and 0.84 for variants with ≤ 50% and ≤ 75% of normal activity, respectively (Figure 5A, left panel). The prediction accuracy of gain‐of‐function variants was significant but lower (AUC_ROC_ of 0.71 and 0.69 for variants with ≥ 125% and ≥ 150% of normal activity). At the individual gene level, SSEP performed well across transporters (Figure 5A, right). Performance was particularly strong for OCT1 (AUC_ROC_ = 0.91) and SERT (AUC_ROC_ = 0.93), both of which benefited from DMS‐based fine‐tuning and remained high for MATE2‐K (AUC_ROC_ = 0.93), CNT3 (AUC_ROC_ = 1.00), and OATP2B1 (AUC_ROC_ = 0.90). In contrast, OATP1B1 (AUC_ROC_ = 0.72) showed comparatively lower performance, likely due to limited training coverage relative to its strong substrate dependence. When benchmarked against widely used variant effect predictors (Figure 5B), SSEP outperformed general‐purpose models, with a mean AUC of 0.912 compared with 0.889 for AlphaMissense and 0.876 for FATHMM‐XF. Furthermore, extension to other genes including ABC transporters and drug metabolizing enzymes showed that SSEP performed well overall, confirming model transferability (Figure S3). Together, these results demonstrate that SSEP accurately classifies functional variants across diverse transporters and enzymes, highlighting the value of incorporating substrate‐specific information into pharmacogenomic prediction models.

### 3.5 Feature Exploration for the Prediction of Substrate‐Specific Variant Effects

**FIGURE 6:**
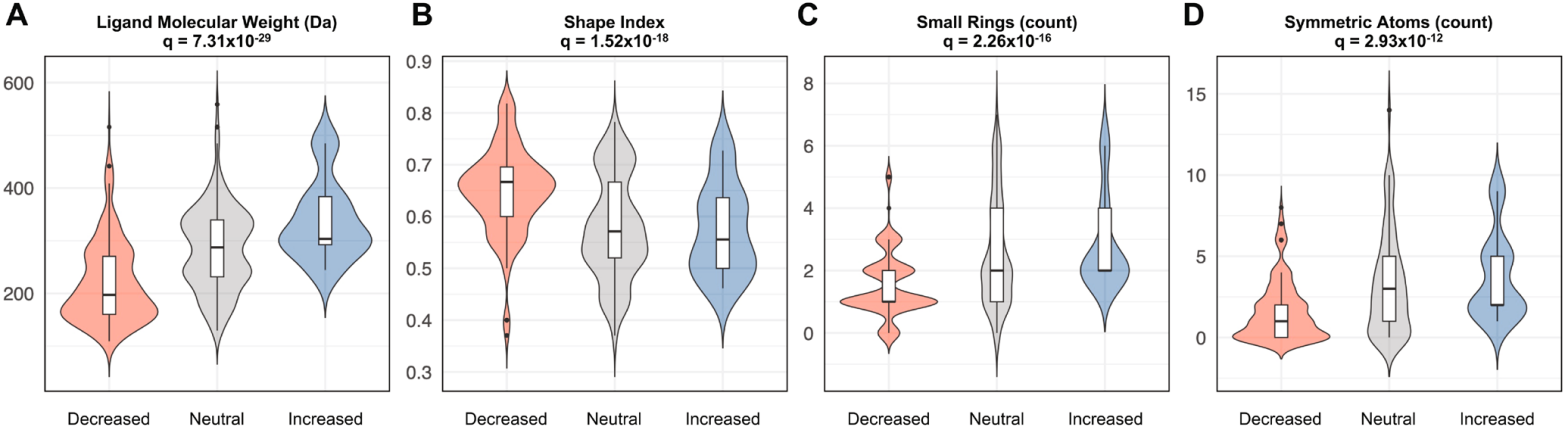
Top features associated with substrate‐specific uptake categories. Variant–substrate pairs were stratified by their impact on substrate transport into decreased (≤ 50%), neutral (50%–200%), and increased (> 200%). Ligand molecular weight (A), shape index (B), small ring count (C), and symmetric atom counts (D) significantly explained variant‐substrate activity. Violin plots show per‐group distributions with overlaid boxplots (white fill). Colors denote uptake category. Statistics for feature labels denote false discovery rate‐adjusted *q*‐values.

To explore properties that discriminate the substrate‐specific variant effects, we grouped variant‐substrate activities into three groups (decreased, neutral, and increased). Statistical comparison across these groups revealed several features that significantly distinguished predicted functional outcomes. Ligand molecular weight showed the strongest association (*q* = 7.31 × 10^−29^), with higher values more frequently observed among increased‐activity variant–substrate pairs (Figure 6A). Mechanistically, these results suggest that larger ligands form more extensive contact interfaces within the transporter binding pocket, making their transporter more sensitive to conformational changes induced by amino acid substitutions. In contrast, shape index, which captures three‐dimensional complexity and rigidity, was negatively associated with increased activity (*q* = 1.52 × 10^−18^), indicating that simpler topology or less rigid ligands may adapt more readily to conformational variation (Figure 6B). Ligands containing a greater number of small rings also tended to enhance activity (*q* = 2.26 × 10^−16^), consistent with compact rings acting as structural anchors that maintain favorable interactions within mutation‐altered binding pockets, allowing efficient binding without introducing steric hindrance (Figure 6C). Similarly, higher molecular symmetry was associated with increased activity (*q* = 2.93 × 10^−12^), reflecting the advantage of multiple equivalent binding orientations that facilitate stable binding even in altered transporter conformations (Figure 6D). Collectively, these results indicate that transport of larger, symmetric ligands with simple topology and compact ring motifs is less likely to be impacted by transporter variants, highlighting key chemical determinants that modulate the substrate‐specific functional effects of transporter variants.

### 3.6 Real‐World Application in UK Biobank

To assess the clinical applicability of SSEP, we associated genetic and prescription data from the UK Biobank. Specifically, we focused on the OCT1 substrate metformin. In prior studies, metformin response had been reported to be associated with the p.M420del variant [19, 42], whereas a later meta‐analysis of a large diabetes cohort did not find associations between common functional OCT1 variants and metformin efficacy [43]. Here, we observed a significant association with the OCT1 variant burden weighted by metformin‐specific SSEP scores with maintenance dose across 9513 patients prescribed metformin (Table 1). Lower metformin‐specific SSEP scores correspond to reduced OCT1 transport activity. Accordingly, lower values of SSEP‐weighted burden reflect a higher cumulative loss‐of‐function burden across OCT1 variants. Thus, a one‐unit decrease in the SSEP score, corresponding to lower predicted OCT1 activity, is associated with an approximately 30 mg/day higher prescribed metformin maintenance dose when adjusted for age at prescription, sex, ethnic group, and BMI (*p* = 0.033). This pattern is consistent with an efficacy‐driven dose titration mechanism, whereby reduced OCT1‐mediated transport may lead to diminished glucose‐lowering efficacy and compensatory dose escalation among individuals who tolerate continued therapy. In contrast, a substrate‐agnostic weighting using the mean in vitro activity across OCT1 substrates showed no association (*p* = 0.78). In a head‐to‐head comparison restricted to OCT1 variants with available in vitro metformin transport data (*n* = 8), a burden score weighted by experimentally measured metformin uptake showed no association with maintenance dose (*β* = 2.44 mg/day, *p* = 0.93), whereas the corresponding metformin‐specific SSEP‐weighted score remained significantly associated (*β* = 28.87 mg/day, *p* = 0.047; Table 1). To further evaluate performance, we performed rare‐variant association tests for metformin response (Table 2). Compared with other widely used variant effect predictors, SSEP produced the strongest signal (SKAT‐O *p* = 0.026; burden *p* = 0.014), followed by ESM1b (SKAT‐O *p* = 0.036; burden *p* = 0.019) and PolyPhen‐2 (SKAT‐O *p* = 0.046; burden *p* = 0.025). Together, these analyses suggest that substrate‐specific weighting through SSEP might capture meaningful variant effects in large‐scale biobank data that are not identified when using substrate‐agnostic aggregation.

**TABLE 1:** Association between OCT1 variant burden and metformin maintenance dose.

Burden (weight definition)	Variant set	*β* (mg/day) [95% CI]	*p*
SSEP (metformin‐specific activity)	11 variants (in vitro available)	30.05 [−2.3 ~ 57.8]	**0.0338**
Mean activity across OCT1 substrates	11 variants (in vitro available)	−7.68 [−60.5 ~ 45.1]	0.7755
SSEP (metformin‐specific activity)	8 variants (metformin in vitro available)	28.87 [−0.42 ~ 57.32]	**0.0467**
In vitro metformin activity	8 variants (metformin in vitro available)	2.44 [−51.98 ~ 56.87]	0.9299

*Note:* Significant values with *p* < 0.05 are indicated in bold. Linear‐model estimates (*β*) show the change in average daily dose (mg/day) per one‐unit increase in burden (sum of allele dosages × weight across rare variants), adjusted for age at prescription, sex, ethnic group, and BMI. Weights: SSEP uses metformin‐specific SSEP score weights; Mean activity across OCT1 substrates uses a nonsubstrate‐specific mean in vitro activity per variant. In total, 9513 metformin users from UK Biobank were analyzed.

**TABLE 2:** Rare variant association test results for metformin.

Predictor	Damaging variants	SKAT‐O	Burden
SSEP	31	**0.0261**	**0.0143**
ESM1b	32	**0.0356**	**0.0190**
Polyphen2 HDIV	29	**0.0456**	**0.0248**
AlphaMissense	13	0.0554	**0.0353**
MetaLR	21	0.0569	**0.0314**
SIFT4G	36	0.0598	**0.0326**
PROVEAN	46	0.0683	**0.0373**
SIFT	43	0.0692	**0.0374**
MetaSVM	22	0.0764	**0.0429**
Polyphen2 HVAR	23	0.0816	**0.0459**
CADD	40	0.0837	**0.0458**
ClinPred	29	0.1118	0.6599
LRT	35	0.1232	0.0692
MetaRNN	27	0.1499	0.6685
DEOGEN2	24	0.1515	0.0897
BayesDel noAF	24	0.1996	0.1231
MutationTaster	42	0.2203	0.1283
REVEL	10	0.3136	0.2176
BayesDel addAF	19	0.4865	0.3458
FATHMM	6	0.4990	0.4175

*Note:* Significant values with *p* < 0.05 are indicated in bold. Summary of association results for rare damaging variants identified by different variant effect predictors. Columns show the number of damaging variants included in the analysis and *p*‐values from SKAT‐O and burden tests. Lower *p*‐values indicate stronger evidence for association with metformin response.

## 4 Discussion

Drug transporters from the SLC [44] and ABC [45] superfamilies harbor a plethora of genetic variations and it is long known that many of these variants exert substrate‐dependent effects. Nevertheless, existing variant effect predictors cannot consider substrate specificity and assign each variant a single substrate‐agnostic activity score. In this study, we developed SSEP to model how transporter variants alter drug uptake in a substrate‐dependent manner. By combining curated uptake assays with DMS data and applying optimized machine‐learning frameworks, the resulting SSEP framework accurately captured substrate‐specific variant effects. The model reproduced in vitro functional patterns of different transporter variants and successfully identified substrate‐specificity when applied to an independent experimental dataset. Furthermore, the model identified an association between damaging OCT1 variants and metformin doses in the UK Biobank—a finding that was lost when using substrate‐agnostic scoring. Thus, by integrating ligand descriptors with structural features, SSEP can capture substrate‐dependent variability, which correlates with real‐world prescribing patterns.

DMS datasets for OCT1 [33] and SERT [34] provided quantitative activity scores across nearly all amino acid substitutions in the respective transporters, thereby establishing a baseline of global transporter functionality. While these data alone do not capture substrate specificity, incorporating DMS scores during fine‐tuning improved model generalizability and reduced overfitting. Uptake assays across multiple substrates provided the substrate‐level resolution necessary for specificity learning. This strategy of combining comprehensive DMS data for single substrates with multisubstrate uptake data for selected variants is expected to become increasingly scalable as more functional genomics data become available.

The ability to generate accurate substrate‐specific predictions has clear translational implications. Variants such as OCT1 p.M420del or p.R61C are functionally neutral for some substrates but cause strongly reduced uptake for others. Current pharmacogenetic classifications label such variants as either “neutral” or “reduced function”, overlooking this complexity. By providing drug‐specific predictions, SSEP enables a more granular understanding of variant function. The benefit is evident from association studies of the variant landscape of *SLC22A1* with metformin maintenance dose in the UK Biobank. Only when substrate‐specificity was considered for each variant did we observe a significant association, whereas no trends were evident when the average activities across OCT1 substrates were used. Given the wide range of prescribed daily doses of immediate release formulations of metformin that typically vary between 500 and 2550 mg [46], these results incentivize to test whether *SLC22A1* genotype‐guided titration of metformin could reduce the time to reach stable dosing without increasing adverse events. Similar benefits may extend to other substrates with pronounced substrate‐specific variant effects, such as sumatriptan or morphine and its prodrugs.

Beyond known compounds, SSEP is designed to generalize to previously uncharacterized substrates through its explicit incorporation of molecular descriptors. Analyses stratified by substrate class and chemical‐space similarity indicate higher prediction reliability for novel substrates that are chemically similar to compounds represented in the training data, providing a practical confidence estimate for new‐substrate predictions (Figure S4). This property supports the potential application of SSEP in early drug development, where transporter liability and variant sensitivity may need to be assessed before extensive experimental characterization.

Several limitations should be considered. First, the size of the pretraining dataset remains modest compared to the diversity of human transporter variation, resulting in drug‐dependent offsets. For instance, SSEP scores were consistently higher for sumatriptan‐variant pairs than for metformin, suggesting that the model learned global, ligand‐driven trends but lacked sufficient information to fully capture position‐specific effects. This likely reflects sparse multisubstrate annotations linking local residue context to substrate‐specific functional outcomes. Second, structural modeling remains constrained by template quality and docking accuracy, which may not fully capture the conformational flexibility or induced fit [47]. Incorporating molecular dynamics simulations can at least partially address this concern but this approach is currently not sufficiently scalable for thousands of variants. Third, transporter‐specific performance estimates should be interpreted cautiously for sparsely represented transporters, where limited substrate diversity may inflate performance metrics. Fourth, as the curated in vitro data were derived from studies reporting relative uptake with individual substrate concentrations not consistently documented, interpretability in terms of *K*_m_ and *V*_max_ remains limited. Finally, validation using UK Biobank prescription data reflects real‐world practices rather than controlled pharmacokinetic measurements, and analysis beyond metformin was limited by low prescription counts or incomplete electronic medical records.

Looking ahead, expanding functional datasets to include more transporters, substrates, and variant types will be meaningful to increase model generalizability. Integrating protein dynamics and increasing coverage of multi‐substrate functional assays will provide richer structural and biochemical context [48]. Particularly, the continued growth of large‐scale functional genomics resources, including additional transporter DMS datasets, will provide valuable training data. Future extensions of SSEP could also incorporate kinetic parameters, such as *V*_max_ or *K*_m_, as such data become available, enabling more mechanistic dissection of substrate‐specific variant effects. Replication in independent cohorts, particularly, across diverse populations, will be essential to establish clinical utility [49]. Should validation and replication efforts confirm that SSEP predictions can add value for guiding tailored prescribing of transporter substrates, then integration of SSEP into pharmacogenomic decision‐support systems could enable variant‐ and drug‐specific recommendations.

In summary, we developed the first computational substrat‐specific variant effect predictor for pharmacogenetic transporter variants that integrates multi‐layered biochemical and structural information. By moving beyond substrate‐agnostic classifications, SSEP represents a step toward a new paradigm that offers the potential to refine precision pharmacogenomics by tailoring drug therapy to the unique interaction between variant and substrate.

## Author Contributions

Y.P., Y.Z., M.,X., and V.M.L. wrote the manuscript. Y.P., M.X., and V.M.L. designed the research. Y.P., A.T.N., and Y.Z. performed the research. Y.P. analyzed the data.

## Funding

The authors' laboratory received funding from the Swedish Research Council (Grant 2021‐02801, 2023‐03015, and 2024‐03401), the European Research Commission Consolidator Grant 3DMASH (101170408), the European Union's Horizon Europe program NEMESIS (101137405), by the SciLifeLab and Wallenberg National Program for Data‐Driven Life Science (WASPDDLS22:006), the Robert Bosch Foundation, Stuttgart, Germany, the Deutsche Forschungsgemeinschaft (DFG, German Research Foundation) under Germany's Excellence Strategy—EXC 2180‐390900677, and the National Research Foundation of Korea (NRF) grant funded by the Korea government (MSIT; RS‐2023‐00209528).

## Conflicts of Interest

V.M.L. is cofounder, CEO, and shareholder of HepaPredict AB, as well as cofounder and shareholder of Shanghai Hepo Biotechnology Ltd. The other authors declare no conflicts of interest.

## Supporting information


**Data S1:** cts70592‐sup‐0001‐DataS1.docx.

**Table S1:** Comprehensive list of transporter variant‐substrate pairs included in SSEP model training.

**Table S2:** Comprehensive list of transporter variant‐substrate pairs included in SSEP model evaluation.

**Table S3:** List of PDB protein–ligand complex structures used for model training.

**Table S4:** Overview of features extracted for model training.

**Figure S1:** Hyperparameter optimization results.
**Figure S2:** Effect of DMS fine‐tuning on model performance.
**Figure S3:** Per‐gene distribution of average AUC_ROC_ values for enzyme genes across SSEP and 40 substrate‐agnostic prediction models.
**Figure S4:** Relationship between substrate chemical space and SSEP predictive reliability.
