# Supplementary material for: Feed‐Forward Deep Neural Networks Predict Substrate‐Specific Effects of Transporter Variants to Explain Drug Response Variability
Source: Clin Transl Sci. 2026 May 8;19(5):e70592. doi: 10.1111/cts.70592 (PMC13156069; doi:10.1111/cts.70592)
Supplement: Supplementary file 1 — Data S1: cts70592‐sup‐0001‐DataS1.docx. [file CTS-19-e70592-s006.docx]

**Feed-forward deep neural networks for predicting substrate-specific effects of transporter variants to explain drug response variability**

Yoomi Park, Yitian Zhou, Ming Xiao, Anne T. Nies, Volker M. Lauschke

**Supplementary Methods**

***Mutant structure modelling and ligand docking***

***Ligand curation and 3D preparation****.* Primary ligand structures were retrieved from PubChem using the PUG-REST API ^1^. When a compound name was not resolvable automatically, the record was curated manually by sanitizing SMILES and converting to 3D with RDKit ^2^. For cases with an available PDB ligand, the co-crystal coordinates were used directly after preparation.

***Wild-type template complex assembly.*** Experimentally determined transporter structures were curated from the PDB (Supplementary Table S1). For each target, we (i) enumerated available outward-facing PDB entries mapped by UniProt, (ii) split biological units into chain-specific receptor files, and (iii) removed crystallographic waters and extraneous cofactors. Co-crystallized ligands were isolated per chain and used to define candidate binding pockets by nearest-neighbor distance to the reference ligand. When multiple structures were available for a transporter, non-redundant high-quality chains were retained and carried forward as receptor templates. When a co-crystal ligand was unavailable for the exact receptor chain, we centered the box on the closest bound ligand in a homologous template. Wild-type complexes were generated using AutoDock Vina (v1.1.2).

***Mutant complex modelling.*** Missense and indel variants were built on the wild-type template with MODELLER (v10.5). For single-residue substitutions, we used MODELLER’s mutate pipeline to generate side-chain replacements followed by local relaxation. For deletions, the target sequence was aligned to the template with MUSCLE ^3^, and the alignment was supplied to SWISS-MODEL for target-template mapping ^4^. In cases where PDB files used offset numbering (e.g., chain starts at residue 3), indices were harmonized prior to modelling so that variant positions, features, and annotations matched the UniProt numbering. When experimental PDB coverage was missing at the variant site or a gene had no suitable PDB entry, AlphaFold2 ^5^ was used to model templates. Ligand docking was then performed using Autodock Vina into the predicted wild-type or mutant pockets. For each mutant structure, AutoDock Vina was run and docked poses were converted back to PDB with preserved atom ordering for downstream feature extraction.

***Feature extraction***

***Sequence-level features.*** WT sequences were obtained from FASTA files linked to each PDB entry and mutant amino acid sequences were generated for each variant-substrate pair by introducing string replacements at the designated residue index after harmonizing PDB numbering offsets with UniProt sequence indices (missense variants) or by removing the specified residues from the sequence, followed by realignment to ensure positional consistency with the structural template (indels). Embeddings for each mutant sequence were then generated using the pretrained ESM-2 transformer protein language model ^6^.

***Variant effect prediction scores.*** Variants were annotated with a comprehensive set of in silico prediction scores using ANNOVAR ^7^. For predictors not natively supported in ANNOVAR, specifically PROVEAN and ESM1b, scores were computed manually using locally installed versions of tools ^8, 9^. For OCT1 p.F244A, missing scores were imputed with the mean of the corresponding score across all annotated variants to retain the complete feature set for model training. No imputation was performed for any of the other variants.

***Biochemical and structural descriptors.*** Residue-level physicochemical properties were computed for each mutant position, including volume, molecular weight, hydropathy, aromaticity, charge, and other basic descriptors. Structural stability changes were estimated with FoldX ^10^, using both BuildModel and Stability commands to calculate ΔΔG values (mutant - wildtype) as indicators of mutation-induced destabilization.

***Ligand features.*** DataWarrior (v6.1.0) was used to calculate global molecular descriptors such as molecular weight, cLogP, cLogS, and surface area measures ^11^, while RDKit was applied to compute atomistic and geometric descriptors, including Labute ASA, topological polar surface area (TPSA), number of rotatable bonds, aromatic ring counts, sp³ fraction, and hydrogen bond donor/acceptor counts.

***Environmental and protein-ligand interaction features.*** Local residue environment descriptors were generated with RDKit, and protein-ligand interactions were profiled using PLIP ^12^. Features included residue charge, counts of polar/apolar/charged neighbors, and radial distribution functions (RDFs) of residue-ligand and residue-protein contacts across distance bins. Residues were annotated as part of a binding site if the minimum heavy-atom distance to the ligand was less than 6 Å. In addition, an independent quantitative distance feature was included, defined as the shortest heavy-atom distance between the mutated residue and the bound ligand.

***Hyperparameter optimization and machine-learning model construction***

We built two substrate-specific effect prediction models: the Substrate-Specific Effect Predictor for single nucleotide variants (SSEP-SNV) and the Substrate-Specific Effect Predictor for universal variants including indels (SSEP-UV). SSEP-SNV was applied whenever complete per-variant feature annotations were available. When such annotations were incomplete or unavailable (e.g., for indels or novel variants lacking annotations), SSEP-UV was used instead, relying on universally available sequence features together with structural and physicochemical descriptors. Both models were implemented as multi-input feed-forward deep neural networks (DNNs) in TensorFlow. The architecture comprised five input branches corresponding to (i) ESM-2 sequence embeddings, (ii) evolutionary conservation scores, (iii) mutation biophysical descriptors, (iv) ligand physicochemical descriptors, and (v) local structural environment features. Each branch consisted of one or two dense layers with ReLU activation (ESM-2: 256 units; evolutionary: 32; biophysical: 64; ligand: 128; environment: 64) and dropout regularization (rate 0.3). The branch outputs were concatenated, passed through a shared dense layer (128 units, ReLU, dropout 0.3), and connected to a linear output neuron predicting the continuous relative activity for each variant-substrate pair. Models were trained using the Adam optimizer and mean squared error (MSE) loss. Hyperparameter optimization was performed using the pre-training dataset. A grid search across 24 candidate hyperparameter combinations was conducted, varying learning rates, dropout rates, and head layer size. For each combination, the dataset was randomly partitioned into training (85%) and validation (15%) subsets, and training was repeated five times with different random splits. Because pharmacogenetic models are often used to stratify variants by functional impact (e.g., distinguishing disruptive from tolerated effects), rank-preserving predictions are more clinically informative than minimizing absolute error ^13, 14^. Accordingly, model performance was assessed based on the average Spearman correlation coefficient (ρ) between predicted and observed values across the five runs, and the hyperparameter set yielding the highest average ρ was selected for subsequent analyses (Supplementary Figure S1). Prior to model training, feature selection was applied to reduce redundancy. Pairwise Spearman correlations were computed among all features, and one variable was removed from each pair with an absolute ρ≥0.9, retaining only non-collinear predictors. Final model construction was implemented in TensorFlow ^15^. Using the optimized hyperparameter set, SSEP-SNV and SSEP-UV were retrained on the entire pre-training dataset to maximize the use of available data. To provide a single prediction per variant-substrate pair, we built a unified SSEP score by integrating the two trained modules at inference time. When multiple structural models were available for the same variant-substrate pair, individual SSEP scores across PDB structures were averaged to obtain a single representative value for downstream analyses. This unified model was then used for all downstream evaluations.

***Model application using UK Biobank***

***Data extraction and processing.*** Primary care prescription records from the UK Biobank were used to derive metformin-specific maintenance doses ^16^. Drug names were harmonized using a predefined mapping of generic and brand names. Prescriptions corresponding to combination therapies or entries lacking dosage or quantity information were excluded. All prescriptions were normalized by converting dosage units to milligrams and calculating the total prescribed amount per issue date. For each individual, average daily dose was estimated from the five most recent valid prescription intervals, calculated as the prescribed dose multiplied by the dispensed quantity and divided by the number of days until the next prescription ^17^. To capture continuous, regularly prescribed treatments, we set drug-specific refill-interval criteria: at least 7 days between prescriptions to exclude duplicate or acute records, and no more than 6 months between prescriptions to exclude irregular or discontinued use ^18^. To reduce outlier effects, maintenance dose values outside the 5th-95th percentile range were removed.

***Statistical association testing.*** Associations between OCT1 variant burden and metformin maintenance dose were evaluated using linear regression. To focus on functional rare variation, analyses were restricted to variants with a minor allele frequency (MAF) <0.05 in the non-Finnish European (NFE) cohort of gnomAD and to variants classified as functional by each prediction model. For each individual, a variant burden score was calculated as the sum of allele dosages multiplied by a variant‐specific weight. Weights were defined either by (i) metformin‐specific SSEP scores or (ii) the mean *in vitro* activity across all OCT1 substrates. Linear models were fitted to assess the relationship between variant burden and metformin maintenance dose (mg/day), adjusting for age at prescription, sex, ethnic group, and body mass index (BMI). The same modeling framework was applied using burden weights derived from SSEP and a panel of 19 established *in silico* variant effect predictors. To allow direct comparison, all prediction scores were harmonized such that lower values indicated lower predicted OCT1 activity. For predictors of which native scoring orientation was reversed, scores were inverted before analysis. For rare-variant aggregation tests, damaging variants were defined according to each predictor’s damaging threshold. For SSEP, this threshold was empirically optimized. To this end, we first classified variants as damaging or neutral depending on if their observed relative uptake was ≤50% of >50% of wildtype, respectively. Subsequently, we identified the SSEP score cutoff that best discriminated these variants using Youden’s J statistic. This cutoff was subsequently applied to the UK Biobank to label variants as damaging. Two complementary gene-level association tests, SKAT-O and burden testing, were implemented using the R package SKAT ^19, 20^. Both tests used metformin maintenance dose as a continuous outcome and were adjusted for age, sex, ethnicity, and BMI.

**References**

1. Kim, S.*, et al*. PubChem 2025 update. *Nucleic Acids Res*. **53**, D1516-D1525 (2025).

2. RDKit: Open-source cheminformatics. <https://www.rdkit.org>.

3. Edgar, R.C. MUSCLE: multiple sequence alignment with high accuracy and high throughput. *Nucleic Acids Res*. **32**, 1792-1797 (2004).

4. Waterhouse, A.*, et al*. SWISS-MODEL: homology modelling of protein structures and complexes. *Nucleic Acids Res*. **46**, W296-W303 (2018).

5. Jumper, J.*, et al*. Highly accurate protein structure prediction with AlphaFold. *Nature*. **596**, 583-589 (2021).

6. Lin, Z.*, et al*. Evolutionary-scale prediction of atomic-level protein structure with a language model. *Science*. **379**, 1123-1130 (2023).

7. Wang, K., Li, M. & Hakonarson, H. ANNOVAR: functional annotation of genetic variants from high-throughput sequencing data. *Nucleic Acids Res*. **38**, e164 (2010).

8. Choi, Y. & Chan, A.P. PROVEAN web server: a tool to predict the functional effect of amino acid substitutions and indels. *Bioinformatics*. **31**, 2745-2747 (2015).

9. Brandes, N., Goldman, G., Wang, C.H., Ye, C.J. & Ntranos, V. Genome-wide prediction of disease variant effects with a deep protein language model. *Nature genetics*. **55**, 1512-1522 (2023).

10. Schymkowitz, J.*, et al*. The FoldX web server: an online force field. *Nucleic Acids Res*. **33**, W382-388 (2005).

11. Sander, T., Freyss, J., von Korff, M. & Rufener, C. DataWarrior: an open-source program for chemistry aware data visualization and analysis. *J Chem Inf Model*. **55**, 460-473 (2015).

12. Salentin, S., Schreiber, S., Haupt, V.J., Adasme, M.F. & Schroeder, M. PLIP: fully automated protein-ligand interaction profiler. *Nucleic Acids Res*. **43**, W443-447 (2015).

13. Gerdes, H.*, et al*. Drug ranking using machine learning systematically predicts the efficacy of anti-cancer drugs. *Nat Commun*. **12**, 1850 (2021).

14. Livesey, B.J. & Marsh, J.A. Variant effect predictor correlation with functional assays is reflective of clinical classification performance. *Genome Biol*. **26**, 104 (2025).

15. Abadi, M.*, et al*. Tensorflow: A system for large-scale machine learning. *In the Proceedings of the 12th USENIX Symposium on Operating Systems Design and Implementation (OSDI ’16)*. 2016: 265-283.

16. Bycroft, C.*, et al*. The UK Biobank resource with deep phenotyping and genomic data. *Nature*. **562**, 203-209 (2018).

17. McInnes, G. & Altman, R.B. Drug Response Pharmacogenetics for 200,000 UK Biobank Participants. *Pac Symp Biocomput*. **26**, 184-195 (2021).

18. Jani, M., Birlie Yimer, B., Sheppard, T., Lunt, M. & Dixon, W.G. Time trends and prescribing patterns of opioid drugs in UK primary care patients with non-cancer pain: A retrospective cohort study. *PLoS Med*. **17**, e1003270 (2020).

19. Lee, S., Abecasis, G.R., Boehnke, M. & Lin, X. Rare-variant association analysis: study designs and statistical tests. *Am J Hum Genet*. **95**, 5-23 (2014).

20. Wu, M.C.*, et al*. Rare-variant association testing for sequencing data with the sequence kernel association test. *Am J Hum Genet*. **89**, 82-93 (2011).
