# Supplementary material for: Feed‐Forward Deep Neural Networks Predict Substrate‐Specific Effects of Transporter Variants to Explain Drug Response Variability
Source: Clin Transl Sci. 2026 May 8;19(5):e70592. doi: 10.1111/cts.70592 (PMC13156069; doi:10.1111/cts.70592)
Supplement: Supplementary file 4 — Table S3: List of PDB protein–ligand complex structures used for model training. [file CTS-19-e70592-s002.docx]

**Table S3. List of PDB protein-ligand complex structures used for model training.**

| **PDB ID** | **Method** | **Resolution (Å)** | **Ligand** | **Ref** |
| --- | --- | --- | --- | --- |
| **OCT1** | | | | |
| 8ET7 | Cryo-EM | 3.77 | Diphenhydramine | 1 |
| 8ET8 | Cryo-EM | 3.45 | Verapamil | 1 |
| 8JTT | Cryo-EM | 3.87 | Metformin (Outward-occluded) | 2 |
| 8JTS | Cryo-EM | 4.14 | Metformin (Outward-open) | 2 |
| 8JTZ | Cryo-EM | 3.27 | Spironolactone | 2 |
| **SERT** | | | | |
| 7LIA | Cryo-EM | 3.3 | Cholesterol | 3 |

**Associated references:**

1. Suo Y, Wright NJ, Guterres H, Fedor JG, Butay KJ, Borgnia MJ, Im W, Lee SY. Molecular basis of polyspecific drug and xenobiotic recognition by OCT1 and OCT2. Nat Struct Mol Biol. 2023 Jul;30(7):1001-1011.
2. Zhang S, Zhu A, Kong F, Chen J, Lan B, He G, Gao K, Cheng L, Sun X, Yan C, Chen L, Liu X. Structural insights into human organic cation transporter 1 transport and inhibition. Cell Discov. 2024 Mar 15;10(1):30.
3. Yang D, Gouaux E. Illumination of serotonin transporter mechanism and role of the allosteric site. Sci Adv. 2021 Dec 3;7(49):eabl3857.
