# Supplementary material for: Feed‐Forward Deep Neural Networks Predict Substrate‐Specific Effects of Transporter Variants to Explain Drug Response Variability
Source: Clin Transl Sci. 2026 May 8;19(5):e70592. doi: 10.1111/cts.70592 (PMC13156069; doi:10.1111/cts.70592)
Supplement: Supplementary file 5 — Table S4: Overview of features extracted for model training. [file CTS-19-e70592-s005.docx]

**Table S4. Overview of features extracted for model training.**

| **Category** | **Description** |
| --- | --- |
| **Sequence-level features** | Sequence embeddings derived from the ESM-2 pretrained transformer model |
| **Variant effect prediction scores** | In silico functional prediction scores including SIFT, PolyPhen2 (HDIV/HVAR), CADD, LRT, MutationTaster, FATHMM, REVEL, GERP++, AlphaMissense, SIFT4G, VEST4, MetaSVM, MetaLR, MetaRNN, gMVP, MPC, PrimateAI, DEOGEN2, BayesDel (±AF), ClinPred, LIST-S2, DANN, FATHMM-MKL, FATHMM-XF, GenoCanyon, fitCons, Eigen (raw, PC), PROVEAN, ESM1b |
| **Biochemical & structural descriptors of mutations** | Residue-level physicochemical properties: Volume, molecular weight, heavy atoms, hydropathy, rotatable bonds, sp³ fraction, donor/acceptor status, aromaticity, hydrophobicity, net charge, lumped hydrophobicity, predicted structural stability change |
| **Ligand features** | Global and atomistic ligand descriptors: Molecular weight, cLogP, cLogS, total/relative/polar surface area, drug-likeness, flexibility, shape index, stereocenters, globularity, VDW surface/volume; Labute ASA, TPSA, rotatable bonds, aromatic rings, sp³ fraction, H-bond donors/acceptors, radius of gyration, asphericity, atom counts (non-H, electronegative, aromatic, metal, heteroatoms), ring system descriptors (carbo/hetero, aromatic/non-aromatic, saturated/unsaturated), functional groups (amides, amines, aromatic nitrogens, acidic/basic atoms) |
| **Environmental & protein-ligand interaction features** | Local residue environment and interaction descriptors: Residue charge, counts of polar/apolar/charged neighbors; RDFs of residue-ligand and residue-protein contacts; angular descriptors around ligand; hydrogen bonds, hydrophobic contacts, salt bridges, π-π stacking, cation-π, halogen bonds, binary binding site flag (<6 Å), quantitative distance to bound ligand |
