# Supplementary material for: Feed‐Forward Deep Neural Networks Predict Substrate‐Specific Effects of Transporter Variants to Explain Drug Response Variability
Source: Clin Transl Sci. 2026 May 8;19(5):e70592. doi: 10.1111/cts.70592 (PMC13156069; doi:10.1111/cts.70592)
Supplement: Supplementary file 6 — Figure S1: Hyperparameter optimization results. Figure S2: Effect of DMS fine‐tuning on model performance. Figure S3: Per‐gene distribution of average AUCROC values for enzyme genes across SSEP and 40 substrate‐agnostic prediction models. Figure S4: Relationship between substrate chemical space and SSEP predictive reliability. [file CTS-19-e70592-s001.docx]

**Figure S1. Hyperparameter optimization results.** Bars represent the Spearman correlation coefficient (ρ) for each hyperparameter configuration, defined by the learning rate for pretraining (lrp), learning rate for fine-tuning (lrf), dropout rate (dr), and head dimension (h), with higher values indicating better ranking performance. Orange crosses indicate the corresponding mean squared error (MSE) values, where lower values reflect better predictive accuracy. (A) SSEP-SNV and (B) SSEP-UV

**Figure S2. Effect of DMS fine-tuning on model performance.** Distributions of performance changes (**ΔSpearman ρ** and **ΔMSE**) illustrate the improvement from DMS fine-tuning across all hyperparameter configurations. For each parameter set (24 combinations of learning rates, dropout, and head size), models were trained both with and without DMS fine-tuning using identical data splits (five runs with 85% training and 15% test subsets). The differences in validation performance between the paired runs were then computed as (DMS-tuned − non-DMS). Positive Δρ and negative ΔMSE values indicate better generalization after DMS integration.

**Figure S3. Per-gene distribution of average AUC_ROC_ values for enzyme genes across SSEP and 40 substrate-agnostic prediction models.** Each grey dot represents the mean AUC_ROC_ across substrates with ≥5 variant-substrate pairs for a given predictor and gene. Boxes summarize the distribution of AUC_ROC_ values across all evaluated models. The green triangle marks SSEP. Genes are ordered by SSEP AUC_ROC_ (positives defined as uptake < 50%).

**Figure S4. Relationship between substrate chemical space and SSEP predictive reliability.** (A) Pairwise Euclidean distances between substrates based on ligand molecular features, comparing distances between substrates from different classes (between-class) and within the same class (within-class). (B) Distribution of per-substrate predictive performance stratified by substrate class. Each box represents the distribution of prediction performance across variants for substrates within the indicated class. (C) Relationship between substrate distance to the training chemical space and predictive consistency for substrates not included in model training. Each point represents a novel substrate with sufficient variant coverage (more than 5 variants).
